# A Pay Hospital for London

**Published:** 1903-01-10

**Authors:** 


### A PAY HOSPITAL FOR LONDON.

The Home Hospital movement originated through a letter written by Sir Henry Burdett on April 4th, 1877, which appeared in the *Standard* of April 6th, and was supported in a leading article in the same paper on April 9th, 1877. In this letter Sir Henry advocated the formation of an association for the purpose of founding a hospital replete with every possible comfort and provision for the privacy and proper treatment of well-to-do patients, to which admission should be by payment alone. In consequence of the publicity thus obtained, much general attention was directed to the subject, and articles in support of the movement were published in the medical and other newspapers. On May 4th, 1877, a deputation of public, city, and medical men waited on the Lord Mayor and presented a memorial on the subject, which resulted in a public meeting being held at the Mansion House on June 27th, 1877. Medical and lay memorials were extensively signed by the leading members of the medical staff of all the metropolitan hospitals, including the then presidents of the Royal College of Physicians, London, the Royal College of Surgeons, the General Medical Council, the late Sir William Gull, Sir James Paget, Sir Andrew Clark, Sir Richard Quain, and upwards of two hundred other leading members of the profession, as well as by the leading managers of the metropolitan hospitals of eminence. The lay memorial signatories included the Bishops of London, Guildford, and Winchester, Cardinal Manning, several members of Parliament, Judges, members of the Bar, leading clergymen, and other influential gentlemen of various professions and callings. In the result the Home Hospitals Association for Paying Patients was incorporated in 1878. A sum of upwards of £20,000 was contributed by those interested in the movement, and after overcoming many difficulties, the chief one being to find suitable premises in the west district of London which could be occupied for the purpose, the Master of the Rolls, Sir George Jessel, having reluctantly decided against any leasehold property being held for the Association, a freehold house was purchased in Fitzroy Square, and the first Home Hospital was opened by the Duke of Northumberland and dedicated by the late Bishop of Winchester on June 27th, 1880. Subsequently the Association acquired the freehold of the neighbouring house and the lease of other adjacent premises. After 22 years it became necessary last autumn to practically rebuild the Home Hospital known as Fitzroy House, Fitzroy Square.

### Twenty-Two Years' Work.

Fitzroy House has been conducted throughout on a system which provides that each patient admitted shall be charged but little over the cost price for his accommodation, and shall pay and be attended by his own doctor. It is the opinion of many eminent members of the profession that the management of this hospital has been so uniformly good that, despite the long period of its existence and its, in some degree, falling behind present-day requirements, Fitzroy House has always been regarded as a boon by many of them and also by middle-class patients from all parts of the Empire. A further reason for the popularity of Fitzroy House is that nowhere else in London could patients obtain the same accommodation and care for so small a cost. From first to last 5,376 patients have been under treatment in Fitzroy House, and in 875 cases they have been accompanied by a friend during their residence there. The number of doctors in whose care these patients have been may be gathered from the fact that, taking the number of medical practitioners in attendance each year, we arrive at a grand total of 1,476 practitioners, including practically all the leading medical men of the day. Fitzroy House has always shown a slight margin of profit, though the management have endeavoured to keep down the cost to the patients so as to arrive each year at a result which should merely avoid the occurrence of a debt.

### A Modern Pay Hospital.

It was essential that Fitzroy House should be reconstructed and largely rebuilt, so as to provide a perfectly equipped modern hospital for middle-class patients, containing thirty beds and every modern hospital appliance. Had Fitzroy House not fulfilled its mission by meeting a felt want, the founder and the other gentlemen who have been associated with him in the management, chief amongst them being the late and present Dukes of Northumberland and Mr. Frederick Cox, would have been justified in resting content with the results arrived at, and so the Home Hospital might have passed into the limbo of forgotten things. In consequence of the urgent necessity which existed for the reconstruction of the buildings on the freehold site, the management had to consider whether they should go on with the work or not. Naturally after 22 years they inclined to the feeling that they had fulfilled their mission, but the representations made to them by the members of the medical profession who have found Fitzroy House such a boon to themselves and their patients left them no alternative but to endeavour to raise a sum of £10,000 to enable the necessary rebuilding to be done. In a letter to the founder, Sir Henry Burdett, written in April last, one of the chief medical men in London states: "You have a splendid case to put before the public. The work has been excellent. The principle is as right now as the day you started the Association, but with this advantage: then the public did not know you were right, now they do." Such testimony from a gentleman who has had patients in Fitzroy House for many years continuously is very striking, and the committee felt that it was their duty to ask those who take an interest in the proper treatment of disease and the more intelligent members of the upper middle classes to subscribe this relatively small sum of £10,000, so that Fitzroy House, when reconstructed as a model of all that is best and most up-to-date in hospital matters, may be reopened and launched once more on its useful, necessary career.

Its continuous success is the best proof that the Home Hospital is really a necessity nowadays. During upwards of 20 years of its existence great advances have been made in surgical and medical knowledge. What was deemed adequate then for successful surgery is known not to be so at the present time. More difficult and dangerous cases present themselves at the present day for treatment, and expect relief which they could not obtain formerly. The surroundings of a patient at the time of treatment have been proved by science and experience to be the important things that make for success or otherwise. With a knowledge of these facts the management, who are constantly being called on to take charge of most serious and important cases, feel it would be wrong not to modernise Fitzroy House and to equip it as such a place should be equipped in the light of present knowledge.

### Ten Thousand Pounds.

The fact that the Association has purposely never made a profit out of the many patients that have been through its hands causes the Committee to have confidence that they may appeal to those who have benefited from time to time by the care given them at Fitzroy House to help the Association to raise the sum (£10,000) found to be necessary to bring this Home Hospital up to date. Already £2,000 of the £10,000 has been promised, and the Committee have every confidence that the balance of £8,000, which is still needed, will be speedily forthcoming when it is known that Fitzroy House has been reconstructed and provided with a passenger lift, an operation theatre, thirty single rooms for the reception of patients, and is replete with every modern equipment for facilitating the speedy recovery of surgical cases. In view of the fact that money is urgently needed to meet the architect's certificates, the Committee venture to hope that everyone into whose hands this brief statement may fall will lose no time in sending a contribution to the Building and Reconstruction Fund, c/o Messrs. Cox and Co., Bankers, 16 Charing Cross, S.W.

### Description of the New Pay Hospital.

The new Fitzroy House contains about forty rooms, of which thirty, each with one bed, constitute the wards. There are also dining-room, sitting-room, smoking-room, with several new bathrooms of the most approved type, with ample accommodation for the nursing and executive staff. The kitchen, which is in the basement, is spacious and well-lighted, with a large scullery and a sufficient larder. The office and apartments of the lady superintendent are centrally situated on the first floor. A sanitary tower, which extends to the full height of the building, enables the whole of the lavatories and W.C.'s to be isolated from the wards. The important question as to how to treat the floors so as to render them aseptic was decided in favour of a new material called papyrolith. Papyrolith is a mixture of asbestos, paper pulp, sawdust, magnacite, and chloride of magnesium. It is laid in the form of cement in two thicknesses, and sets to an extremely hard and impervious surface. In some of the wards the floors are of a very light colour, whilst others have been finished in a warm red; the effect produced is artistically attractive and hygienically satisfactory. A model operation room has been provided on the top floor, with a vertical light facing north and a top light in addition. To adapt the theatre for every kind of surgical work, a black roller blind on a new principle has been fixed, which can be drawn down outside the glass to wholly or partially exclude the light, so as to secure a vertical light, or a top light, or artificial light, as may be required by the surgeon in charge of each case. The walls and ceiling of the operation room are lined with opalite. This material is tinted pale sheet glass made in tiles six inches square, on the back of which glass chips are fused. This rough back forms a key for the Keen's cement in which the tiles are bedded. The surface presented by this material is absolutely impervious, and the joints are so fine as to be almost imperceptible. All the rooms have been fitted with electric light, and by the side of each patient's bed is a switch for turning it on or off, and a wall plug for either a surgical lamp or reading lamp, in addition to one for sterilising, heating, actual cautery, and several other purposes. Each floor is provided with an electric apparatus for sterilising or boiling water; food trolleys have been designed for conveying food from the kitchen to the various floors in a food lift. These are made of teak, lined with a non-conducting material known as Cabot's sheathing, which is American eel glass within a casing of asbestos. Inside the Cabot's sheating are sheets of uralite, a material formed of asbestos. Each trolley contains shelves made of perforated aluminium, on which the dinners for the various rooms are placed, and the whole is heated inside by means of an electric plate at the bottom. The electric plate will be charged in the basement and will remain hot for about an hour. New grates for the wards have been designed by Mr. Pridgin Teale, F.R.S., of Leeds, whilst the fittings and new apparatus in the kitchen and sanitary tower are of the most approved and modern types. The passenger lift, which travels from the basement to the fourth floor, and is worked by an electric motor, is of sufficient size to take a patient in an ambulance, a nurse, doctor, and porter, and the time occupied by the whole journey from ground floor to operation-room is less than a minute.

The decorative and artistic effects presented by the interior of the new hospital are charming in many respects. The dining-room has every attribute for comfort and elegance, and a fine ceiling, specially designed. Each of the wards presents a definite scheme of decoration, and all the furniture and fittings have been constructed to harmonise, so that the general effect is most attractive and inviting. The meals of each patient will be served upon elegant trays, which have been specially designed and manufactured for the Pay Hospital by Mr. Charles 'Ffoulkes, of the Pelham Gallery of Arts and Crafts. They present a most brilliant appearance, and are undoubtedly much superior to electro-plate, sheet glass, and every other material previously used for trays. The crockery has been specially selected, and all the tea and dinner services are of the same beautiful design, which has been manufactured by the Royal Copenhagen Porcelain Manufacturing Company. Hygienically and artistically the new Pay Hospital is declared to be probably the most perfect and attractive building for surgical cases which science and experience have yet produced. Further, everything has been done to render this new Pay Hospital as complete as medical requirements demand, whilst the domestic comforts it affords have received no less careful attention. The arrangements and environments throughout are such as to rob the sick-room of depressing or comfortless attributes, and to surround each patient with everything best calculated to encourage the feeling that Fitzroy House must indeed prove a home to all who enter it.

